# Physician Preferences and Prescribing Patterns for Hypertonic and Isotonic Saline Solutions in Upper Respiratory Symptom Management: A Multinational Clinical Practice Survey Across 13 Countries

**DOI:** 10.7759/cureus.95231

**Published:** 2025-10-23

**Authors:** Giorgio W Canonica, Carolina Castillo, Cecilia Bartoli

**Affiliations:** 1 Biomedical Sciences, Humanitas University, Pieve Emanuele, ITA; 2 Medical Affairs, Menarini, Florence, ITA

**Keywords:** comorbidities, nasal saline spray, natural products, respiratory care, seawater-based products

## Abstract

Background: Managing respiratory symptoms in patients with comorbidities presents complex therapeutic challenges due to the risk of drug interactions and contraindications associated with conventional treatments, requiring careful consideration of treatment options. Recent interest in natural therapeutic alternatives, especially seawater-based nasal sprays, reflects evolving physician attitudes and unmet clinical needs. Despite widespread clinical application, multinational data on physician perspectives toward isotonic and hypertonic saline therapies remain limited, particularly in patients with chronic conditions. This study addresses this gap by evaluating physician preferences and prescribing patterns for saline-based nasal therapies across diverse healthcare systems.

Methods: A cross-sectional survey of 398 physicians (specialists 167 (42%), general practice 183 (46%), family practice 48 (12%)) across 13 countries was carried out using online questionnaire methodology. Participants evaluated prescribing patterns and two seawater-based nasal sprays containing exclusively sodium chloride solutions (one isotonic 0.9% and one hypertonic 2.6% concentration) without additional pharmaceutical active ingredients.

Results: Most physicians surveyed reported frequent concerns about prescribing conventional cough, cold, and allergy medications to patients with comorbidities, with 291 (73%) expressing caution regarding pseudoephedrine in cardiovascular or diabetic patients. Seawater-based nasal sprays were highly rated for safety and suitability, with 87-90% of respondents per country emphasizing their appropriateness for use in sensitive populations, including children and pregnant women. Notable international differences emerged, such as a strong preference for corticosteroids in Finland and a pronounced endorsement of nasal saline products in the United Arab Emirates.

Conclusions: These results demonstrate a clear physician consideration for nasal saline and clinically supported respiratory therapies in patients with comorbidities and highlight the necessity of adapting treatment recommendations to local clinical practices and healthcare environments, including saline solutions.

## Introduction

Respiratory tract infections and allergic conditions represent frequent presentations in primary care settings, with antibiotic prescribing rates for acute respiratory infections ranging from 44% in telehealth settings [1] to 80% in traditional practice environments [2]. Acute upper respiratory tract infections include a spectrum of inflammatory conditions affecting multiple anatomical sites within the upper respiratory system, including the nasal cavity, paranasal sinuses, pharyngeal structures, middle ear space, laryngeal tissues, epiglottis, upper airway passages, and bronchial tree [3]. The common cold represents the most prevalent form of upper respiratory infection, characterized as an acute, self-resolving viral illness that primarily targets the upper respiratory tract mucosa. This condition manifests through a constellation of clinical symptoms including paroxysmal sneezing episodes, nasal obstruction and congestion, rhinorrhea, pharyngeal discomfort, persistent cough, low-grade pyrexia, cephalgia, and generalized malaise [3].

The management of respiratory symptoms in patients with comorbidities presents particular challenges, and international variations in prescribing patterns highlight the influence of cultural, educational, and healthcare system factors on clinical decision-making [2,4]. Physicians express significant concern when prescribing conventional treatments to patients with diabetes, cardiovascular disease, and other chronic conditions due to potential drug interactions and contraindications [5]. This has led to increased interest in natural therapeutic alternatives, particularly saline-based nasal therapies [3,6].

Saline nasal irrigation has demonstrated efficacy in multiple clinical contexts and plays a major role in the treatment of persistent rhinosinusitis, offering significant clinical benefits with minimal associated risks [7]. This therapeutic approach functions as a supportive treatment modality that complements both conservative medical therapy and surgical interventions for patients with persistent rhinosinusitis [8]. The therapeutic mechanisms of nasal irrigation are believed to operate through multiple pathways: it helps to reduce mucus viscosity, enhances the natural clearance function of nasal cilia, diminishes inflammatory swelling of nasal tissues, and removes allergens and irritants from the nasal passages and sinus spaces [8]. The osmolality of the commercial compositions of NaCl solution ranges from the physiological level (0.9%) to the hypertonic level with an osmolality of 3% [6]. Hypertonic saline nasal irrigation shows superior symptom improvement compared to isotonic solutions in pediatric allergic rhinitis [9], while isotonic saline irrigation may provide benefits in persistent rhinosinusitis management [10]. Despite these evidence-based benefits, physician adoption patterns and international prescribing variations remain incompletely characterized.

While our study examines physician prescribing preferences, we must recognize the crucial role that community pharmacists play as frontline healthcare providers for patients experiencing respiratory symptoms. The ARIA guidelines for Italy highlight an important reality: pharmacists often represent the first point of contact for patients dealing with rhinitis symptoms [11]. This happens naturally, given their remarkable accessibility and the fact that many pharmacies maintain extended hours compared to traditional medical practices.

Despite increasing clinical use, there remains a paucity of multinational data on physician selection and evaluation of saline-based nasal therapies for upper respiratory symptoms, particularly in individuals with chronic comorbidities. This multinational survey was conducted to address this gap by systematically assessing how physicians from diverse countries approach treatment selection and to characterize patterns and attitudes regarding isotonic (0.9%) and hypertonic (2.6%) seawater-based nasal sprays. The study specifically aimed to (a) evaluate prescribing preferences and perceived safety/efficacy; (b) compare attitudes and practices between countries; and (c) explore physician concerns and decision-making processes in the management of patients with comorbidities, where the safety profile of respiratory therapies is especially relevant.

## Materials and methods

Design and participants

This multinational cross-sectional survey was conducted in May 2025 using established online survey methodology protocols. The study employed English-language questionnaires distributed through random selection of direct email outreach to known internal medicine specialists, general practitioners, and family physicians from Sermo, a global physician network comprising 1.3 million physicians across 150 countries. For sites in countries with non-native English-speaking physicians, instructions emphasized interpretation consistency, and cross-checks for semantic equivalence were conducted. Randomization procedures for participant invitation within the Sermo platform ensured unbiased inclusion. Country quotas ensured comparable geographic representation, with stratification by specialty and years of clinical experience. Eligible participants included practicing physicians spending ≥65% of their time in active patient care. Exclusion criteria included incomplete survey responses and duplicate entries. The final sample comprised 398 physicians across 13 countries: Colombia, Germany, Spain, Finland, France, Ireland, Italy, Lithuania, Mexico, Peru, Saudi Arabia, South Africa, and United Arab Emirates. Participants voluntarily agreed to participate in this market research study and received payment for their participation. The collected data were fully anonymous.

Survey

The questionnaire (all questions are presented as supplementary material in Appendices) assessed multiple domains consistent with established physician survey methodology: demographic and practice characteristics, patient population prevalence for respiratory conditions and comorbidities, current prescribing patterns for nasal sprays and respiratory treatments, concern levels regarding specific medications in comorbid patients, attitudes toward nasal saline products in respiratory care, and product evaluations for two seawater-based formulations.

Participants reviewed standardized profiles for an isotonic aerosol (0.9% salt concentration, containing seawater from Gullman fjord, fortified with marine minerals; indicated for allergic rhinitis management) and a hypertonic aerosol (2.6% salt concentration, with an identical seawater source; designed for nasal congestion relief).

Statistical analyses

Descriptive statistics characterized physician demographics and response patterns. Country-specific comparisons examined international variations in prescribing behaviors and product attitudes. Response frequencies were calculated for Likert-scale items assessing product importance ratings​.

Institutional review board or ethics committee approval was not required for this study, as it constituted market research involving anonymous physician opinions and prescribing practices, falling outside the scope of human subjects research requiring formal ethical oversight.

## Results

All 398 physicians met the inclusion criteria for active patient care engagement. The majority reported over 11 years of clinical experience (57%) and 33% reported between 5 and 10 years of clinical experience (Table 1).

**Table 1:** Years spent in clinical practice per country of the physicians who replied to the survey (n=398) n: number of subjects. All results are presented as n (%)

Country	n	<5 years	5-10 years	11-20 years	>20 years
Overall	398	36 (9)	131 (33)	127 (32)	100 (25)
Colombia	30	2 (7)	17 (57)	9 (30)	2 (7)
Germany	33	2 (6)	12 (36)	16 (48)	3 (9)
Spain	35	2 (6)	8 (23)	9 (26)	16 (46)
Finland	30	1 (3)	3 (10)	4 (13)	22 (73)
France	30	2 (7)	16 (53)	5 (17)	7 (23)
Ireland	30	2 (7)	6 (20)	17 (57)	5 (17)
Italy	30	2 (7)	8 (27)	11 (37)	9 (30)
Lithuania	30	3 (10)	10 (33)	6 (20)	11 (37)
Mexico	30	4 (13)	13 (43)	8 (27)	5 (17)
Peru	30	11 (37)	16 (53)	2 (7)	1 (3)
Saudi Arabia	30	0 (0)	9 (30)	18 (60)	3 (10)
South Africa	30	5 (17)	7 (23)	7 (23)	11 (37)
United Arab Emirates	30	1 (3)	8 (27)	17 (57)	4 (13)

Overall, there was a balanced representation across specialists (42%) and GPs (46%) with a smaller representation of family medicine physicians (12%). There was an overall response rate of 72%. Most countries contributed 30 physicians, with Germany (33) and Spain (35) providing larger samples.

Patient population prevalence for respiratory conditions and comorbidities

Physicians reported substantial patient loads with respiratory conditions. Upper respiratory tract infections affected >25% of weekly patient encounters for 54% of surveyed physicians. Seasonal allergies were also prevalent, with 38% of physicians seeing >25% of patients weekly with this condition. Chronic respiratory conditions showed notable variation: persistent rhinitis affected 5-15% of weekly patients for 36% of physicians, while COVID-related cough remained relatively uncommon (<5% weekly patients for 46% of physicians) (Table 2).

**Table 2:** Responses to the following question: In a typical week, what percentage of your adult patients have the following conditions? (n=398) Results are expressed as number (%) *Due to rounding, row values may not sum to 100%.

Condition	<5%	5-15%	16-25%	>25%
Seasonal rhinitis (%)	32 (8)	88 (22)	127 (32)	151 (38)
Persistent rhinitis (%)	68 (17)	143 (36)	111 (28)	76 (19)
Persistent sinusitis (%)*	103 (26)	135 (34)	96 (24)	60 (15)
COVID cough (%)	183 (46)	96 (24)	76 (19)	44 (11)
Upper respiratory tract infections (%)*	8 (2)	60 (15)	119 (30)	215 (54)

**Figure 1:**
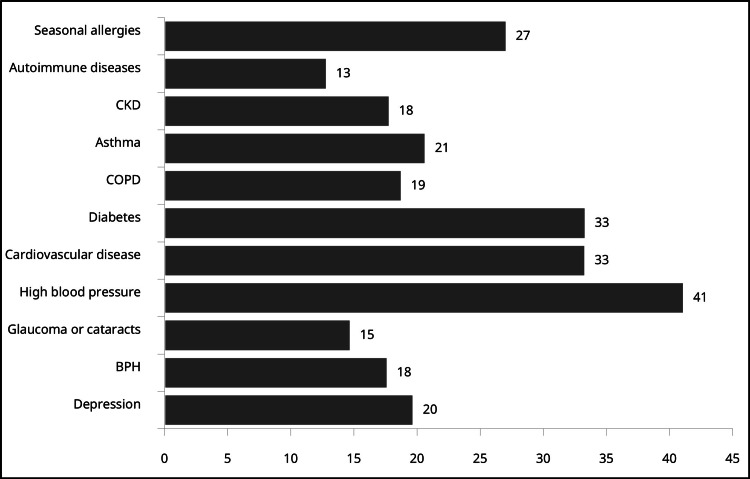
Percentage of adult patients with comorbidities BPH, benign prostatic hyperplasia; CKD, chronic kidney disease; COPD, chronic obstructive pulmonary disease.

Hypertension represented the most common comorbidity (mean 41% of adult patients), followed by diabetes (33% of patients) and cardiovascular disease (33% of patients). Respiratory-specific comorbidities included seasonal allergies (27% of patients) and asthma (21% of patients) (Figure 1).

Current prescribing patterns for nasal sprays and respiratory treatments

The current therapeutic landscape demonstrates clear hierarchies in physician prescribing behavior across different nasal spray categories. Seawater and saline-based formulations command the strongest physician confidence, with two-thirds of practitioners frequently recommending these products and the remaining third using them occasionally. Corticosteroid nasal preparations maintain substantial utilization rates, prescribed regularly by 61% of physicians and intermittently by an additional 38% of practitioners. Nasal sprays containing topical decongestants occupy a less common position in prescribing patterns, with less than half of physicians using them frequently while a similar proportion employs them selectively. Nasal sprays containing antihistamines demonstrate the most conservative usage profile, prescribed regularly by 44% of surveyed physicians, though nearly half utilize them on an occasional basis (Table 3).

**Table 3:** Prescription patterns for nasal sprays and respiratory treatments (n=398) Results are expressed as number (%)

	Never	Sometimes	Often
Nasal sprays containing corticosteroids	4 (1)	151 (38)	243 (61)
Nasal sprays containing topical decongestants	44 (11)	171 (43)	179 (45)
Nasal sprays containing antihistamines	36 (9)	187 (47)	175 (44)
Nasal sprays containing seawater/saline	0 (0)	135 (34)	263 (66)

Concern levels regarding specific medications in comorbid patients

**Figure 2:**
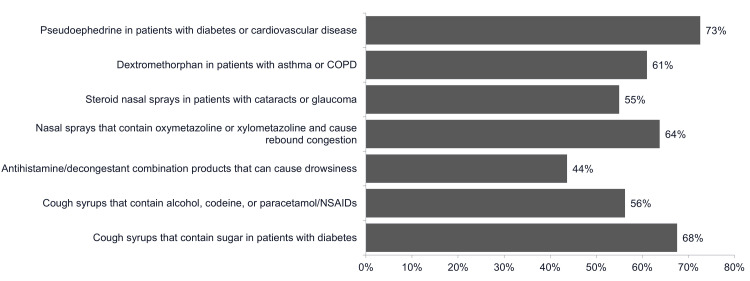
Concern levels regarding specific medications in comorbid patients. Physicians who are somewhat concerned or prefer to avoid (n=398) COPD, chronic obstructive pulmonary disease; NSAIDS, non-steroidal anti-inflammatory drugs.

Clinical decision-making reflects heightened awareness of medication risks in patients with multiple conditions. Pseudoephedrine use in patients with cardiovascular disease or diabetes generates concern among 73% of physicians, reflecting well-documented risks associated with these agents in these vulnerable populations. Rebound congestion phenomena associated with certain nasal decongestants that contain oxymetazoline or xylometazoline concern 64% of physicians. Dextromethorphan usage in patients with asthma or chronic obstructive pulmonary disease generates apprehension among 61% of practitioners, reflecting concerns about respiratory depression in compromised patients (Figure 2).

Attitudes toward nasal saline products in respiratory care

**Figure 3:**
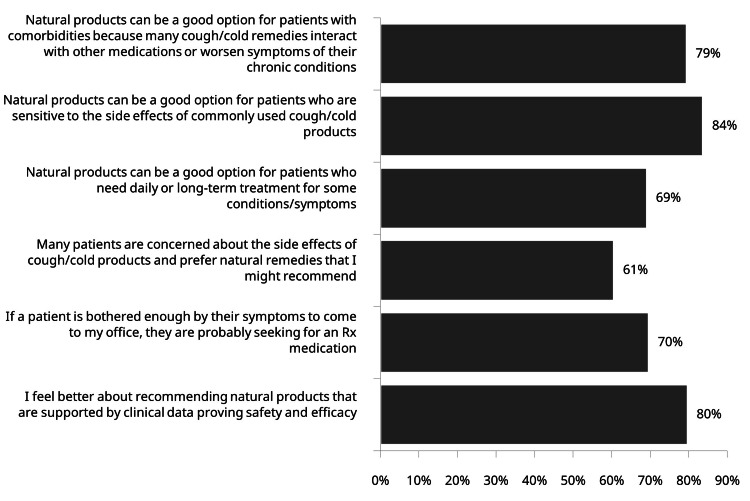
Physician agreement with statements on the use of natural products for managing respiratory symptoms in patients with chronic conditions (n=398)

Physician attitudes toward nasal saline products reveal substantial endorsement across multiple clinical scenarios. Seventy nine percent of practitioners recognize benefits of natural products specifically for patients managing multiple comorbidities, while an even higher proportion (84%) prefer these options for patients particularly sensitive to cold conventional medication side effects. Long-term treatment scenarios prompt natural product consideration among 69% of physicians, and 80% express greater confidence when recommending natural products supported by clinical data proving safety and efficacy (Figure 3).

Product evaluations

**Figure 4:**
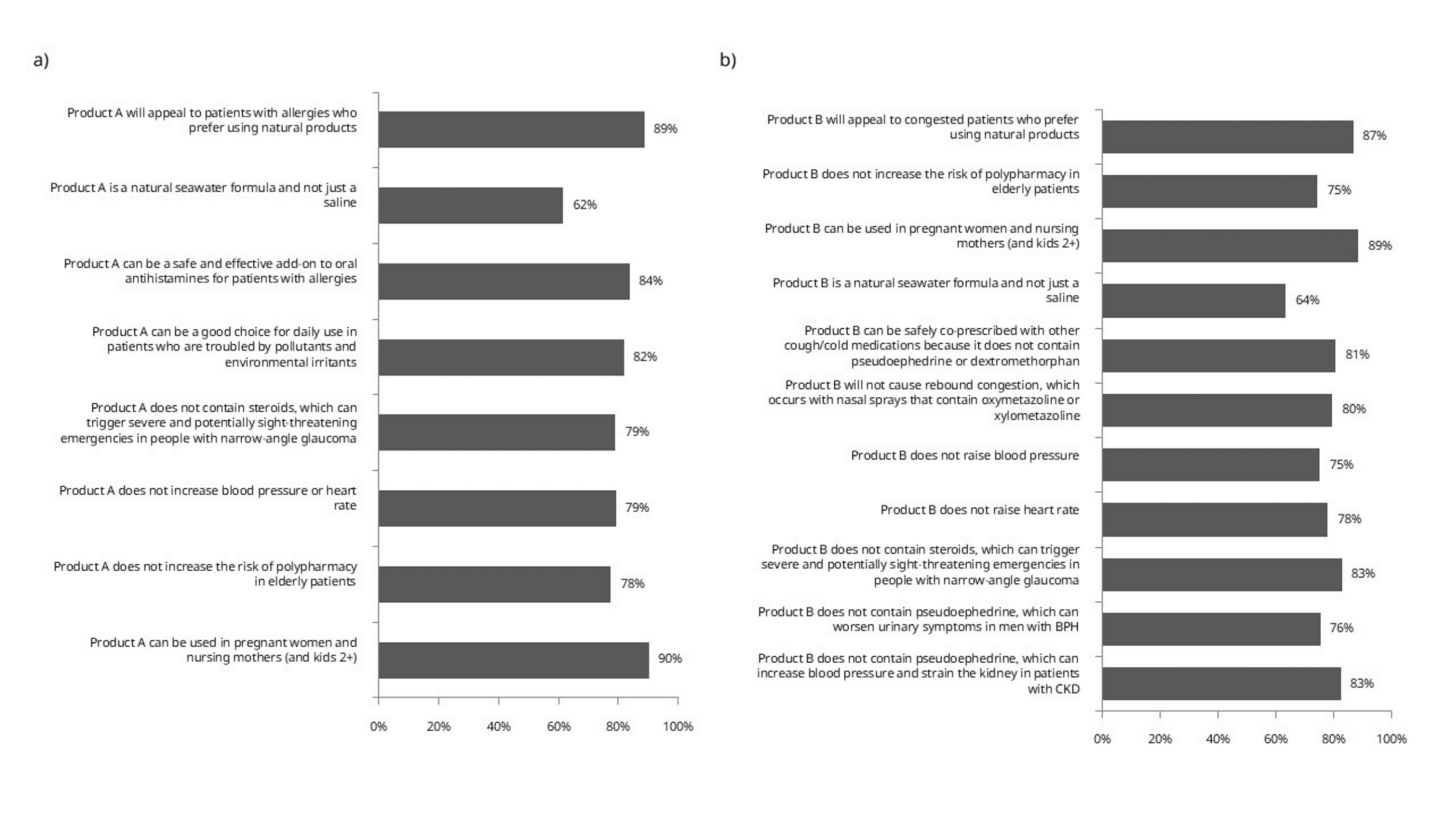
Product evaluations for two seawater-based formulations (n=398) Data represents physician's agreement with the statements. a) Evaluation of product A, corresponding to the isotonic aerosol (0.9% salt concentration, containing seawater from Gullman fjord, fortified with marine minerals; indicated for allergic rhinitis management). b) Evaluation of product B, corresponding to the hypertonic aerosol (2.6% salt concentration, with an identical seawater source designed for nasal congestion relief). BPH, benign prostatic hyperplasia; CKD, chronic kidney disease; COPD, chronic obstructive pulmonary disease; COVID, coronavirus disease.

Safety profiles of isotonic seawater products generate a high physician approval, with 90% reporting that it can be used in pregnancy, nursing, and pediatric use (>2 years old) as important or very important. Physicians report that it will appeal to patients with allergies seeking natural alternatives receives similarly high ratings from nearly 90% of practitioners; 84% report that it can be a safe and effective add-on to oral antihistamines for patients with allergies (Figure 4a).

Hypertonic formulations demonstrate comparable safety endorsement, with 89% of physicians rating pregnancy and pediatric safety as important. 87% of physicians report that it will appeal to congested patients who prefer using natural products. Freedom from pseudoephedrine-related complications earns recognition from 81% of surveyed physicians (Figure 4b).

International variations in prescribing behaviors

Geographic differences in prescribing patterns reveal significant variations in therapeutic approaches across different healthcare systems. Finnish physicians demonstrate remarkably high corticosteroid utilization rates (97%) compared to 61% in the broader international sample (supplementary data).

United Arab Emirates practitioners exhibit high concerns about the use of pseudoephedrine in comorbid patient management with diabetes and cardiovascular disease, with 97% rates compared to 73% overall rates. In this country, 87% of physicians also report that natural products can be a good option for patients with comorbidities because many cold remedies interact with other medications or worsen symptoms of their chronic conditions (Supplementary data). This preference may reflect cultural attitudes toward natural therapeutics, regulatory environments favoring such products, or specific patient population characteristics within United Arab Emirates medical practice. 

## Discussion

This multinational survey of 398 physicians across 13 countries provides clear physicians' support for the prescription of nasal saline products, particularly in patients with comorbidities. The high concern levels regarding conventional treatments (61-73% across different medications), together with strong natural product endorsement (79-84%), highlight significant unmet clinical needs in respiratory care. Seawater-based solutions received very high physician approval, with 87-90% importance ratings for safety profiles during pregnancy, nursing, and pediatric use, while natural product appeal garnered similarly high ratings from nearly 90% of practitioners.

Our findings provide additional evidence of physician enthusiasm for the seawater-based products, which reflects growing recognition of evidence-based natural alternatives in respiratory medicine [3,6]. Here, we document strong physician confidence in these approaches across diverse healthcare systems and practice settings. Previous research has demonstrated that hypertonic saline nasal irrigation shows superior symptom improvement compared to isotonic solutions in allergic rhinitis, while isotonic saline irrigation provides meaningful benefits in chronic conditions [9]. A randomized controlled trial in children with perennial allergic rhinitis demonstrated that 3% hypertonic saline provided greater improvement in total nasal symptom scores than normal saline, particularly for nasal congestion [3]. A comprehensive systematic review and meta-analysis examined nine randomized controlled trials involving 740 patients and provided definitive evidence regarding the comparative effectiveness of hypertonic versus isotonic saline irrigation. The analysis demonstrated that hypertonic saline nasal irrigation brought greater benefits over isotonic saline (standardized mean difference -0.58; 95% confidence interval: -0.76, -0.40) [9,12].

The observed therapeutic preferences reflect clinicians' heightened awareness of safety considerations when treating patients with complex medical histories, particularly those managing multiple chronic conditions [13], where drug interactions and contraindications significantly complicate treatment decisions. Clinical prudence dictates careful evaluation of conventional therapeutic options in pregnancy [14] and comorbid populations, as evidenced by documented cardiovascular risks associated with pseudoephedrine administration [5]. This clinical caution has encouraged increased acceptance of natural therapeutic alternatives, with seawater-based interventions gaining particular favor among healthcare providers due to their favorable risk-benefit profiles.

Our findings regarding nasal spray prescribing patterns present clear hierarchies in physician prescribing behavior, with seawater and saline-based formulations showing the strongest physician confidence. This preference pattern suggests that physicians are increasingly incorporating evidence-based natural alternatives into their treatment algorithms, particularly for patients with complex medical histories or medication sensitivities. The positive physician attitudes toward seawater-based nasal products are aligned with clinical evidence supporting this therapy's effectiveness [8-10].

The survey results indicate that therapeutic guidelines must be tailored to the realities of each country. Prescribing patterns and physician attitudes toward nasal saline products differ between countries, shaped by factors such as regulatory frameworks and clinical traditions. For instance, the high frequency of corticosteroid prescribing among Finnish physicians stands in contrast to the strong preference for natural therapies observed in the United Arab Emirates, reflecting how local policies and incentives influence everyday clinical choices, such as the recommendation to add saline nasal sprays as an add-on therapy for patients with persistent symptoms [15]. These findings point to the need for international recommendations to be adapted to local practice environments, taking into account scientific evidence and established prescribing habits, patient expectations, and the organization of healthcare systems.

Several limitations should be highlighted when interpreting our findings. The online survey methodology, while enabling international reach, may introduce selection bias favoring technology-comfortable physicians. Response bias represents another concern, as physicians interested in respiratory products may be overrepresented. The English-language requirement potentially excluded physicians with limited English proficiency, particularly impacting countries with non-native English speakers. The sample size of approximately 30 physicians per country limits statistical power for country-specific analyses; for this reason, readers are cautioned not to overinterpret national contrasts, as these findings are primarily descriptive and hypothesis-generating rather than confirmatory. While our study focused exclusively on physician perspectives, future research should examine pharmacist attitudes and prescribing patterns for saline-based products, given their role as primary healthcare contacts for many patients with respiratory symptoms [13]. Additionally, the focus on seawater-based products may have attracted physicians with existing natural product interests and approaches. Despite these limitations, our study offers several strengths. First, the multinational design captured perspectives across diverse healthcare systems, providing valuable insights into cross-cultural variations in clinical practice. Second, the high response rate and diverse specialty representation enhance the generalizability of our findings to various clinical settings. Third, the inclusion of detailed product evaluations provides specific insights into the features of nasal saline products that physicians value most, which can inform future product development and clinical guidelines. Finally, the high response rate and diverse specialty representation enhance the generalizability of our findings to various clinical settings.

## Conclusions

Physician preferences and attitudes towards saline-based nasal therapies for upper respiratory symptoms in comorbid populations were assessed across 13 countries, capturing a uniquely broad and internationally relevant perspective. The findings indicate a strong inclination among practitioners to recommend natural products, such as seawater-based nasal sprays, particularly where conventional medications raise safety concerns. This observed trend underscores a growing clinical shift towards safer, non-pharmaceutical options in respiratory care. However, it is crucial to emphasize that these insights are derived solely from perceptual and self-reported data, limiting the ability to establish causal or clinical efficacy relationships. Additionally, country-specific differences were observed, likely influenced by local healthcare practices and regulatory environments, but should be interpreted cautiously given methodological constraints.

The principal take-home message is that, while this research provides valuable hypothesis-generating information with broad international applicability, further studies, including interventional trials and real-world outcome research, are needed to rigorously document the effectiveness and optimal integration of saline-based therapies for complex patient populations. The study’s strength lies in its multinational scope and cross-disciplinary participation, whereas its limitation is the reliance on physician perceptions rather than objective clinical endpoints.

## Figures and Tables

**Figure 5:**
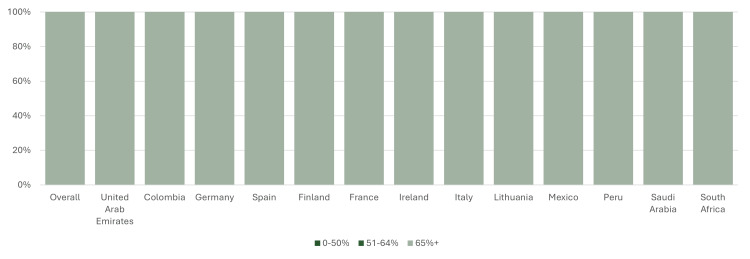
What percentage of your time is spent on patient care?

**Figure 6:**
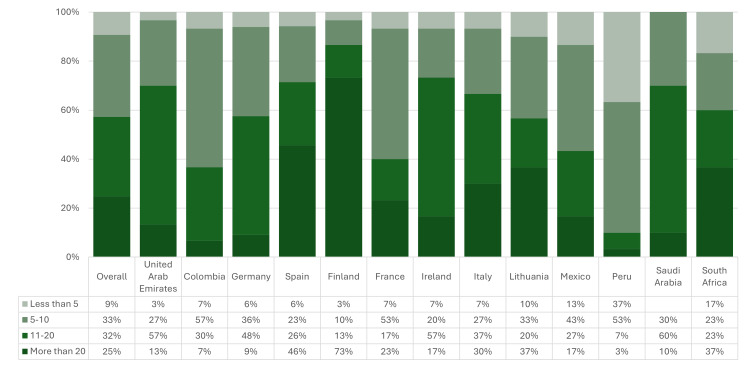
How many years have you been in practice?

**Figure 7:**
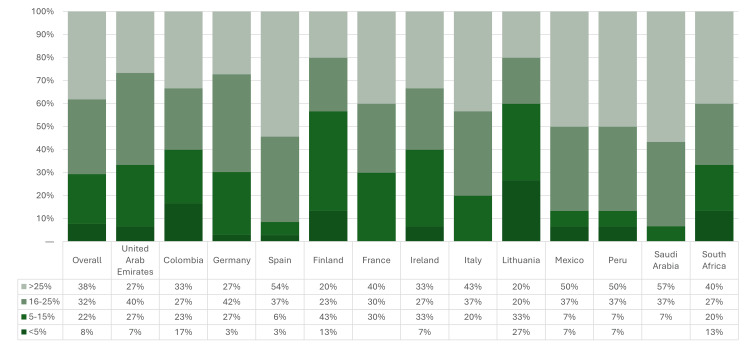
In a typical week, what percentage of your adult patients have the following conditions? - Seasonal rhinitis

**Figure 8:**
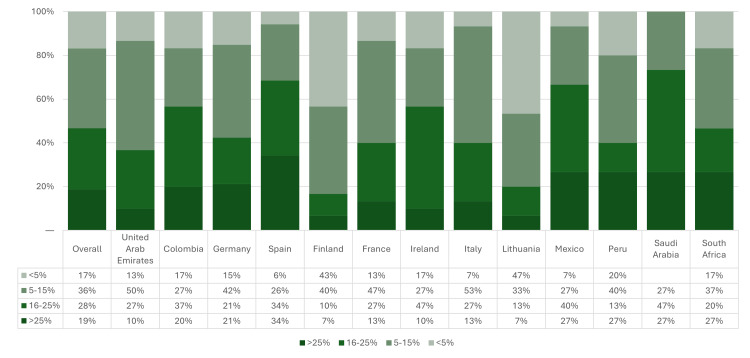
In a typical week, what percentage of your adult patients have the following conditions? - Persistent rhinitis

**Figure 9:**
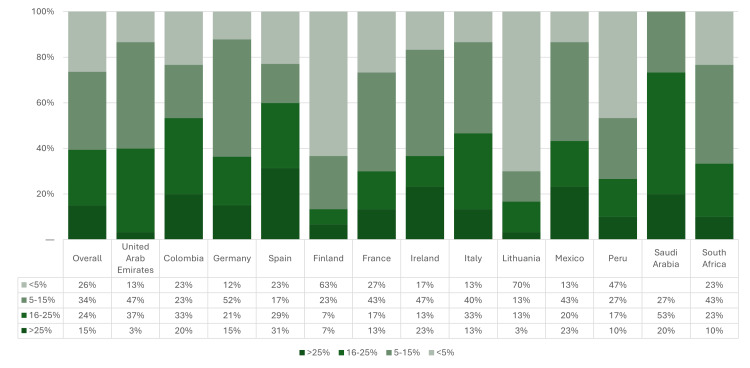
In a typical week, what percentage of your adult patients have the following conditions? - Persistent sinusitis

**Figure 10:**
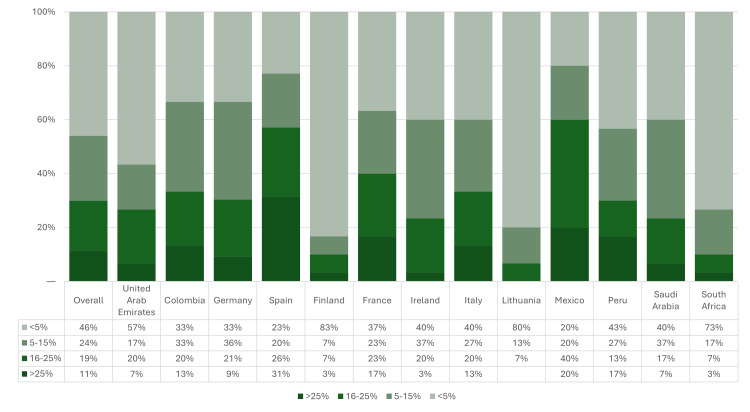
In a typical week, what percentage of your adult patients have the following conditions? - COVID cough

**Figure 11:**
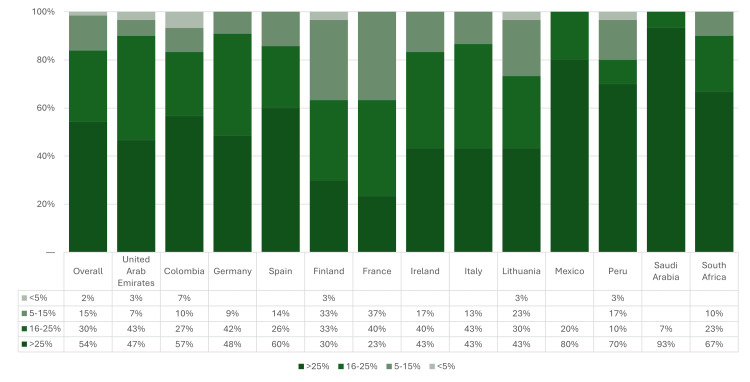
In a typical week, what percentage of your adult patients have the following conditions? - Upper respiratory tract infections

**Figure 12:**
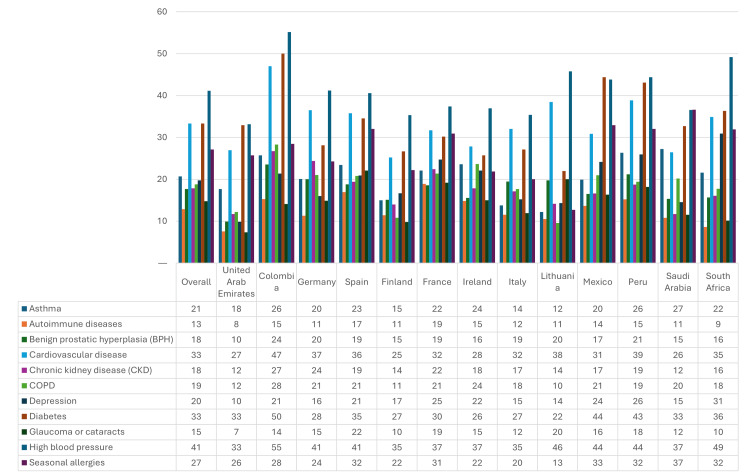
What percentage of your adult patients have the following chronic conditions?

**Figure 13:**
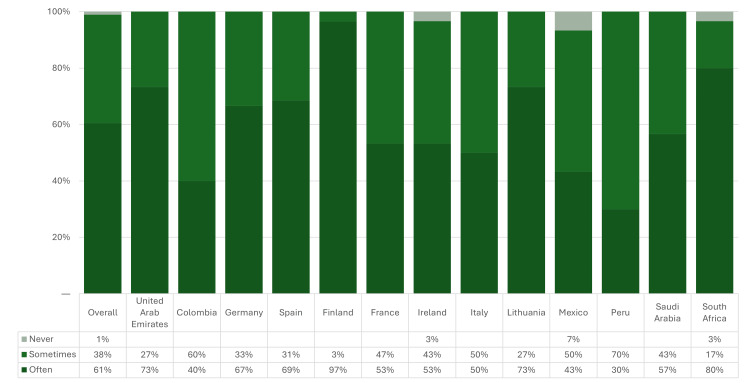
How often do you prescribe or recommend the following products? - Nasal sprays containing corticosteroids

**Figure 14:**
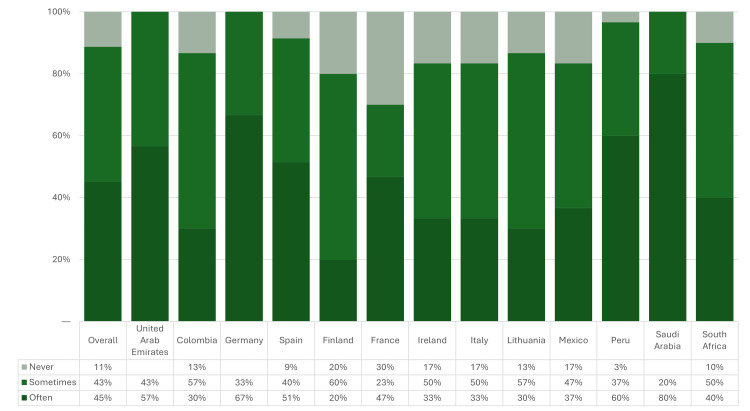
How often do you prescribe or recommend the following products? - Nasal sprays containing decongestants

**Figure 15:**
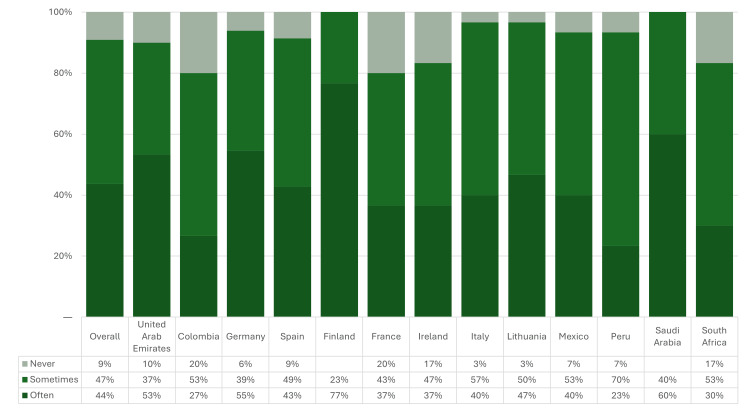
How often do you prescribe or recommend the following products? - Nasal sprays containing antihistamines

**Figure 16:**
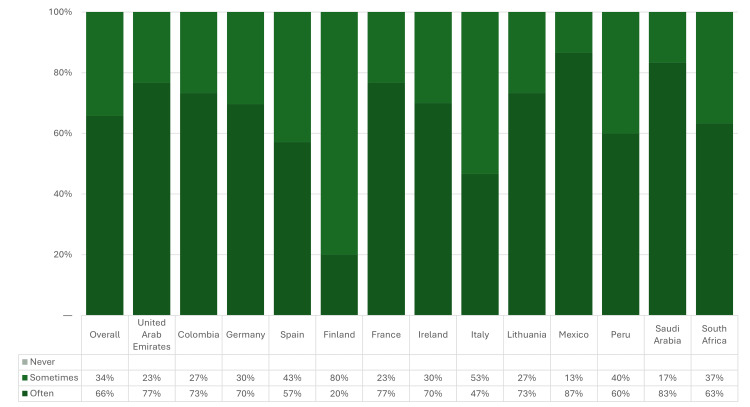
How often do you prescribe or recommend the following products? - Nasal sprays containing seawater/saline

**Figure 17:**
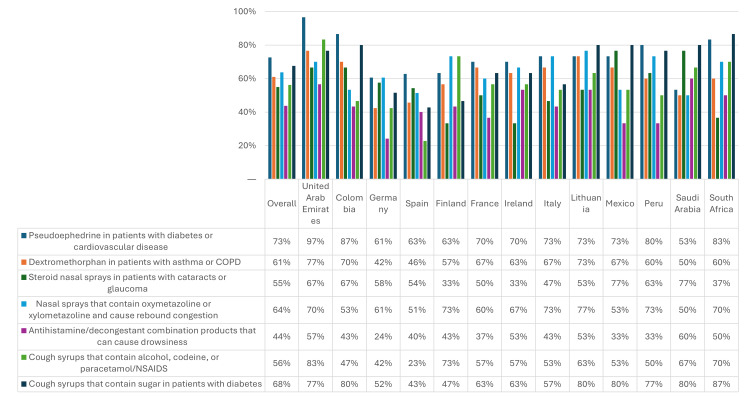
We would like to gauge your level of concern/caution when recommending or prescribing some medications to certain patients. Which best describes your level of concern?

**Figure 18:**
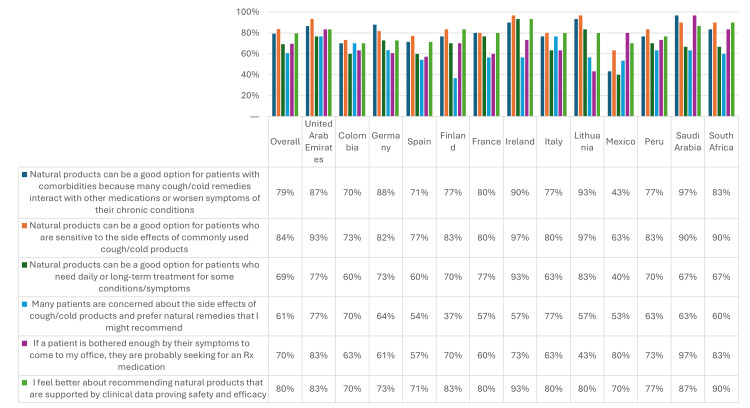
Thinking about patients with chronic conditions who need relief from cough, cold, sore throat, or congestion symptoms, how do you respond to the following statements?

**Figure 19:**
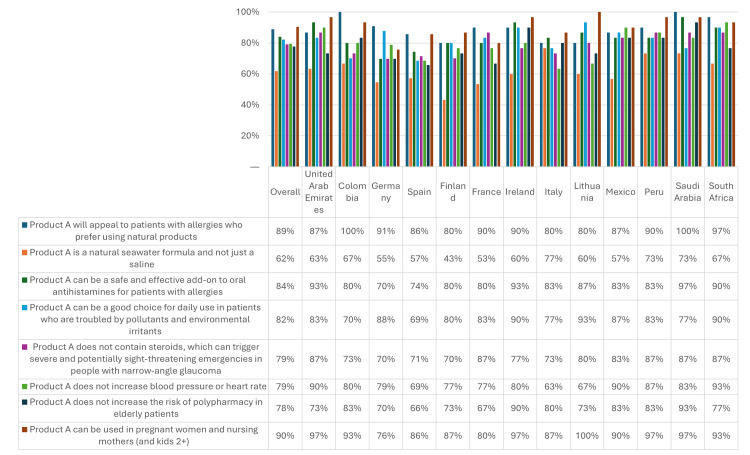
Quixx delicate Profile (Product A) - Based on this brief product profile, how would you respond to the following statements about Product A?

**Figure 20:**
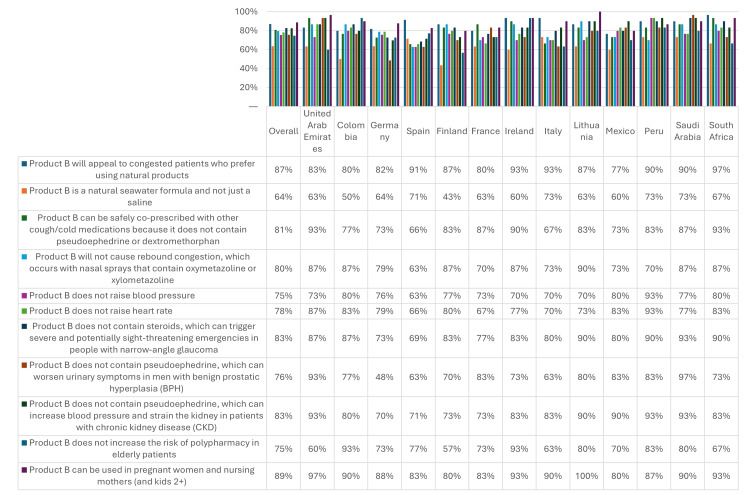
Quixx forte Product Profile (Product B) - Based on this brief product profile, how would you respond to the following statements about Product B?

## Appendices

Complete survey

1.     What percentage of your time is spent on patient care?

2.     How many years have you been in practice?

3.     In a typical week, what percentage of your adult patients have the following conditions? - Seasonal rhinitis

4.     In a typical week, what percentage of your adult patients have the following conditions? - Persistent rhinitis

5.     In a typical week, what percentage of your adult patients have the following conditions? - Persistent sinusitis

6.     In a typical week, what percentage of your adult patients have the following conditions? - COVID cough

7.     In a typical week, what percentage of your adult patients have the following conditions? - Upper respiratory tract infections

8.     What percentage of your adult patients have the following chronic conditions?

9.     How often do you prescribe or recommend the following products? - Nasal sprays containing corticosteroids

10.  How often do you prescribe or recommend the following products? - Nasal sprays containing decongestants

11.  How often do you prescribe or recommend the following products? - Nasal sprays containing antihistamines

12.  How often do you prescribe or recommend the following products? - Nasal sprays containing seawater/saline

13.  We would like to gauge your level of concern/caution when recommending or prescribing some medications to certain patients. Which best describes your level of concern?

14.  Thinking about patients with chronic conditions who need relief from cough, cold, sore throat, or congestion symptoms, how do you respond to the following statements?

15.  Quixx delicate Profile (Product A) - Based on this brief product profile, how would you respond to the following statements about Product A?

16.  Quixx forte Product Profile (Product B) - Based on this brief product profile, how would you respond to the following statements about Product B?
